# Survival benefit of living-donor liver transplantation in patients with a model for end-stage liver disease over 30 in a region with severe organ shortage: a retrospective cohort study

**DOI:** 10.1097/JS9.0000000000000634

**Published:** 2023-08-10

**Authors:** Seung Hyuk Yim, Deok Gie Kim, Minyu Kang, Hwa Hee Koh, Mun Chae Choi, Eun Ki Min, Jae Geun Lee, Myoung Soo Kim, Dong Jin Joo

**Affiliations:** Department of Surgery, The Research Institute for Transplantation Yonsei University College of Medicine, Seoul, South Korea

**Keywords:** deceased organ shortage, end-stage liver disease, living-donor liver transplantation, treatment intention, waitlist mortality

## Abstract

**Background:**

The benefits of living-donor liver transplantation (LDLT) in patients with a high Model for End-stage Liver Disease (MELD) score (who have high waitlist mortality) are unclear. Regional availability of deceased-donor organs must be considered when evaluating LDLT benefits. The authors aimed to compare the survival benefit of intended-LDLT to awaiting deceased-donor liver transplantation (DDLT) in patients with a MELD score greater than or equal to 30 in a region with severe organ shortage.

**Materials and methods:**

This retrospective review included 649 patients with a MELD score greater than or equal to 30 placed on the liver transplantation waitlist. They were divided into intended-LDLT (*n*=205) or waiting-DDLT (*n*=444) groups based on living-donor eligibility and compared for patient survival from the time of waitlisting. Post-transplantation outcomes of transplant recipients and living donors were analyzed.

**Results:**

Intended-LDLT patients had higher 1-year survival than waiting-DDLT patients (53.7 vs. 28.8%, *P*<0.001). LDLT was independently associated with lower mortality [hazard ratio (HR), 0.62; 95% CI, 0.48–0.79; *P*<0.001]. During follow-up, 25 patients were de-listed, 120 underwent LDLT, 170 underwent DDLT, and 334 remained on the waitlist. Among patients undergoing transplantation, the risk of post-transplantation mortality was similar for LDLT and DDLT after adjusting for pretransplantation MELD score (HR, 1.86; 95% CI, 0.73–4.75; *P*=0.193), despite increased surgical complications after LDLT (33.1 vs. 19.4%, *P*=0.013). There was no mortality among living-donors, but 4.2% experienced complications of grade 3 or higher.

**Conclusions:**

Compared to awaiting DDLT, LDLT offers survival benefits for patients with a MELD score greater than or equal to 30, while maintaining acceptable donor outcomes. LDLT is a feasible treatment for patients with a MELD score greater than or equal to 30 in regions with severe organ shortages.

## Introduction

Highlights

- Living-donor liver transplantation (LDLT) may benefit patients in regions with organ shortage, but its use in patients with a high Model for End-stage Liver Disease (MELD) score requires further investigation.
- LDLT improves survival in patients with a MELD score greater than or equal to 30, compared to awaiting deceased-donor liver transplantation.
- LDLT should be considered as feasible treatment option for patients with a MELD greater than or equal to 30.

Living-donor liver transplantation (LDLT) is a viable option for patients with end-stage liver disease facing a shortage of deceased-donor livers^1^. LDLT offers a timely and reliable option for patients who have a high risk of mortality while on the waitlist in regions with severe organ shortages, such as many Asian countries^2,3^. When considering LDLT, one must appropriately balance the risks and benefits for both the recipient and the potential donor ^4,5^. Therefore, it is important to perform LDLT only in situations when positive outcomes are expected with a reasonable degree of certainty for the recipient, as well as the donor^6^.

The Model for End-stage Liver Disease (MELD) is a scoring system used to predict the mortality rate of patients awaiting liver transplantation (LT) and is used as a standard for liver allocation in many countries^7–9^. The MELD score is significantly associated with outcomes after LT^10^. In 2002, the New York State Committee on Quality Improvement in Living Liver Donation recommended prohibiting LDLT in patients with a MELD score greater than 25 because of the possibility of a futile transplantation^11^. There is also controversy regarding the safety of partial grafts in patients with a high MELD score^12,13^.

Recent studies have shown that LDLT performed at experienced centers can produce outcomes comparable to those achieved with deceased-donor LT (DDLT)^14–18^. However, most studies comparing LDLT and DDLT have only compared patients who actually underwent these procedures, without accounting for patients who died while on the waitlist. To accurately assess the benefits of LDLT, it is necessary to compare LDLT to DDLT with its waiting period. Wong *et al*.^19^ recently presented data from a single center in Hong Kong (which faces a shortage of deceased-donor livers) and demonstrated a survival benefit of LDLT in patients with a MELD score greater than or equal to 25 when analyzed on an intention-to-treat basis. It is crucial to note that the benefit of LDLT in patients with high MELD may vary by region, based on the deceased-donor pool. Thus, we conducted this study to compare the survival benefit of LDLT to waiting for DDLT in patients with a MELD score greater than or equal to 30, which is virtually the minimum requirement for deceased-donor liver allocation in South Korea (Supplemental Digital Content 1, http://links.lww.com/JS9/A842).

## Methods

### Study participants

Between September 2005 and December 2021, 713 patients with a MELD score greater than or equal to 30 who were on the waitlist for LT at our center were retrospectively reviewed. We excluded 64 patients with hepatocellular carcinoma (HCC) with distant metastasis, any other malignancy diagnosed within 5 years, or significant deterioration in health status within 2 days of placement on the waitlist.

Of the 649 patients included in the study, those with potential living donors were categorized as intended-LDLT group and those with no potential living-donor at the time of enlisting were categorized as waiting-DDLT group. Additionally, patients who passed more than 90 days from the time of waitlisting to the time of LDLT were regarded as not having an intention to perform LDLT and were therefore categorized into the waiting-DDLT group. These were the initial categories based on treatment intention. The patients were also placed in three categories based on their actual treatment: LDLT, DDLT, and waitlist-only, which consisted of patients who were not eventually allocated for LT. Some patients from the intended-LDLT group ended up receiving DDLT because they were also listed for DDLT. Therefore, when they were allocated to deceased-donor liver, we proceeded with DDLT. Conversely, some patients in the waiting-DDLT group finally underwent LDLT. Although, they initially did not have an eligible living-donor at the time of waitlisting, they were subsequently able to find a living-donor. If a patient in the intended-LDLT group did not undergo LDLT, the reason for this decision was recorded.

All study procedures were conducted in accordance with the Declaration of Helsinki, as revised in 2013. The institutional review board of Hospital approved the study (4-2022-0913), and patient consent for this study was waived because of its retrospective design. This retrospective study has been reported in line with the strengthening the reporting of cohort, cross-sectional, and case–control studies in surgery (STROCSS) criteria^20^ (Supplemental Digital Content 12, http://links.lww.com/JS9/A853).

### LDLT evaluation

We reviewed the medical histories and laboratory results of all recipients and donors. A series of evaluations, including dynamic computed tomography, MRI (including magnetic resonance cholangiopancreatography), and liver fibrosis scans, were conducted to investigate the anatomy of the liver. The volume of the graft was estimated using computed tomography volume analysis. After calculating the graft-to-recipient weight ratio, anatomical variations of the portal vein, hepatic artery, and hepatic duct were assessed. According to the results, we selected appropriate donors and grafts for each recipient.

### Variables and study endpoints

We calculated the uncapped MELD score instead of the capped MELD score because clinical outcomes differ when the MELD score is greater than or equal to 40^21^. We also calculated the MELD score increase, which was defined as the increase in MELD score during the month before the day of waitlisting. The MELD score increase was divided into three categories: less than 15, greater than or equal to 15, and initial MELD score at presentation greater than or equal to 30. Information regarding each patient’s underlying liver disease and the presence of viable HCC was collected. Acute liver failure (ALF)^22,23^, acute-on-chronic liver failure (ACLF), and chronic liver failure were identified as the indications for LT. ACLF was graded from 0 to 3^24^. Chronic kidney disease (CKD) was defined as a diagnosis of CKD within 3 months before the patient reached a MELD score of 30, an estimated glomerular filtration rate less than 60 ml/min/1.73 m^2^, or the presence of proteinuria. Patients with serum creatinine greater than or equal to 1.5 mg/dl and no CKD history were classified as having hepatorenal syndrome (HRS)^25^. Patients with heart failure, coronary artery disease, valvular heart disease, cerebral hemorrhage, and cerebrovascular accident were considered to have cerebrovascular disease. Pretransplantation hospitalization days were recorded, with a particular focus on ICU stays.

The primary study endpoint was patient survival of the intended-LDLT and waiting-DDLT groups. Survival was calculated from the date of waitlisting until the date of patient death or 31 December 2021 (the end of the follow-up period). The actual transplantation survival rate of patients who underwent LT was the secondary endpoint. It was calculated from the date of LT until the date of patient death or 31 December 2021.

In addition, information regarding several outcomes of LT was collected and recorded, including duration of post-transplantation hospital stay, rate of retransplantation, presence of complications, and occurrence of rejection. We graded complications using the Clavien–Dindo classification system, with major complications defined as grade ≥3 complications^26^.

### Statistical analysis

Data are presented as a number (proportion) for categorical variables and as a mean with SD or median with interquartile range (IQR) for continuous variables. To determine the significance of intergroup differences, the Fisher’s exact test was used for categorical variables, and the *t*-test or Mann–Whitney *U* test was used for continuous variables. The Kaplan–Meier method was used to analyze patient survival rates, and the log-rank test was used to compare survival rates between groups. When comparing the survival rate of the three post-transplantation groups post-hoc, the Bonferroni method was used to calculate the *P*-value. We used Cox regression analysis with the backward stepwise method to identify variables independently associated with survival in the treatment intention population, as well as in the post-transplantation groups.

Inverse probability of treatment weighting (IPTW) was conducted to reduce selection bias between the intended-LDLT and waiting-DDLT groups^27^. Propensity scores were extracted from binomial logistic regression employing all baseline variables and utilized to calculate weights. All calculations were performed using R 4.2.0 for MacOS (http://cran.r-project.org, R Foundation for Statistical Computing). *P*-values <0.05 were considered statistically significant.

## Results

**Figure 1:**
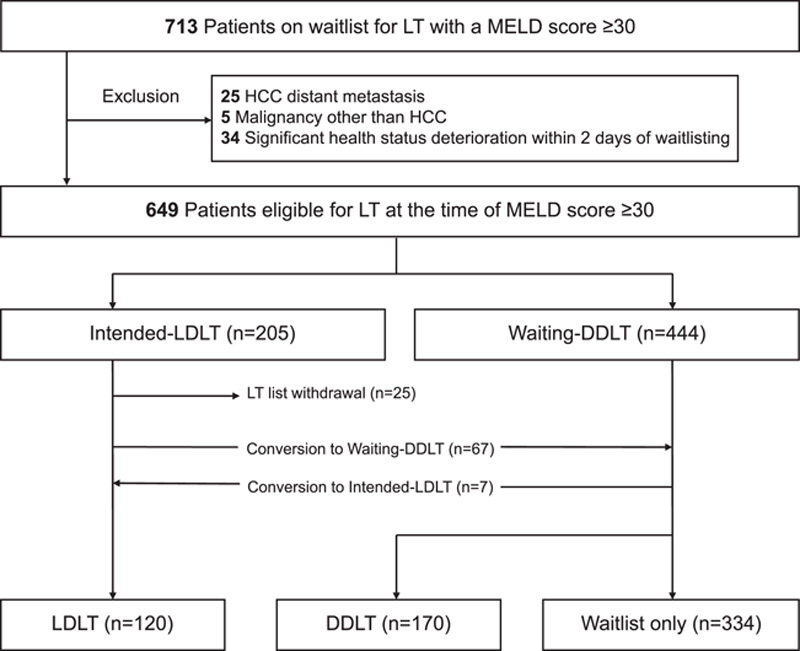
Study flowchart. DDLT, deceased-donor liver transplantation; HCC, hepatocellular carcinoma; LDLT, living-donor liver transplantation; LT, liver transplantation; MELD, Model for End-stage Liver Disease.

A total of 649 patients with a MELD score greater than or equal to 30 at the time of waitlisting were included in the study. The intended-LDLT group included 205 (31.6%) patients, whereas the Waiting-DDLT group included 444 (68.4%) patients. The flowchart of the intended and actual treatment is depicted in Fig. 1. Among 205 patients in the intended-LDLT group, 25 (12.2%) patients were removed from the waitlist because of deteriorating health status, 20 (9.8%) were allocated to DDLT, and 47 (22.9%) did not receive LT because of donor eligibility issues, such as incompatible donor, donation withdrawal, refusal from the Korean Network for Organ Sharing, or other reasons (Supplemental Digital Content 2, http://links.lww.com/JS9/A843). A total of 113 (55.1%) patients in the Intended-LDLT group underwent LDLT. In the waiting-DDLT group, 7 (1.6%) patients converted to the LDLT group while awaiting LT, 150 (33.8%) patients underwent DDLT, and 287 (64.6%) patients remained on the waitlist. Of the 649 patients in the study, 120 underwent LDLT, 170 underwent DDLT, and 334 patients remained on the waitlist.

### Baseline characteristics on waitlisting day

Baseline characteristics according to the intended treatment group are summarized in Table 1. There were no differences between intention-to-treat groups for sex; ABO blood type; type of underlying liver disease; ICU stay prior to waitlisting; presence of pneumonia; or presence of major organ failure involving the brain, circulatory system, or respiratory system. MELD scores were similar between groups, with no differences in proportions of patients with MELD scores of 30–34, 35–39, or greater than or equal to 40.

**Table 1:** Baseline characteristics at the time of waitlisting in patients with a Model for End-stage Liver Disease score greater than or equal to 30.

Variables	Intended-LDLT (*n*=205)	Waiting-DDLT (*n*=444)	*P*
Age, year	50 (43–58)	54 (46–62)	<0.001
Sex, male	137 (66.8)	319 (71.8)	0.227
ABO type			0.236
* *A or B	130 (63.4)	257 (57.9)	
* *AB	16 (7.8)	52 (11.7)	
* *O	59 (28.8)	135 (30.4)	
Underlying liver disease			0.084
* *Alcoholic	43 (21.0)	109 (24.5)	
* *HBV	90 (43.9)	211 (47.5)	
* *HCV	9 (4.4)	25 (5.6)	
* *HAV	10 (4.9)	11 (2.5)	
* *Cryptogenic	23 (11.2)	54 (12.2)	
* *Autoimmune	15 (7.3)	16 (3.6)	
* *Other	15 (7.3)	18 (4.1)	
MELD			0.774
* *30–34	138 (67.3)	310 (69.8)	
* *35–39	43 (21.0)	89 (20.0)	
* *≥40	24 (11.7)	45 (10.1)	
Hepatocellular carcinoma	34 (16.6)	153 (34.5)	<0.001
Liver failure type			0.010
* *ACLF 0	23 (11.2)	33 (7.4)	
* *ACLF 1	13 (6.3)	49 (11.0)	
* *ACLF 2	86 (42.0)	192 (43.2)	
* *ACLF 3	51 (24.9)	132 (29.7)	
* *ALF	32 (15.6)	38 (8.6)	
Hepatorenal syndrome	85 (41.5)	250 (56.3)	0.001
Organ failure, brain	24 (11.7)	71 (16.0)	0.188
Organ failure, circulatory system	45 (22.0)	107 (24.1)	0.616
Organ failure, respiratory system	23 (11.2)	53 (11.9)	0.894
Chronic kidney disease	4 (2.0)	39 (8.8)	0.002
Cardiovascular disease	3 (1.5)	30 (6.8)	0.008
ICU stay before waitlisting, patients	47 (22.9)	106 (23.9)	0.869
MELD score increase in the prior 1 month			0.012
* *<15	32 (15.6)	113 (25.5)	
* *≥15	39 (19.0)	88 (19.8)	
Initial score ≥30	134 (65.4)	243 (54.7)	
Sepsis	14 (6.8)	55 (12.4)	0.046
Pneumonia	16 (7.8)	37 (8.3)	0.941

Data are presented as number (percentage) or median (interquartile range).

ACLF, acute-on-chronic liver failure; ALF, acute liver failure; DDLT, deceased-donor liver transplantation; HAV, hepatitis A virus; HBV, hepatitis B virus; HCV, hepatitis C virus; LDLT, living-donor liver transplantation; MELD, Model for End-stage Liver Disease.

Some baseline characteristics differed between groups. The intended-LDLT group was significantly younger than the waiting-DDLT group [median age, 50 years (IQR, 43–58) vs. 54 years (IQR, 46–62), *P*<0.001] and had a lower proportion of patients with HCC (16.6 vs. 34.5%, *P*<0.001), HRS (41.5 vs. 56.3%, *P*=0.001), CKD (2.0 vs. 8.8%, *P*=0.002), and sepsis (6.8 vs. 12.4%, *P*=0.046). Compared to the waiting-DDLT group, the intended-LDLT group included a significantly higher proportion of patients with ALF (15.6 vs. 8.6%) and ACLF 3 (24.9 vs. 29.7%). The proportion of patients with an initial MELD score greater than or equal to 30 was higher in the intended-LDLT group, and the proportion with a MELD score increase less than 15 was higher in the waiting-DDLT group (*P*=0.012).

### Patient survival according to treatment intention

**Figure 2:**
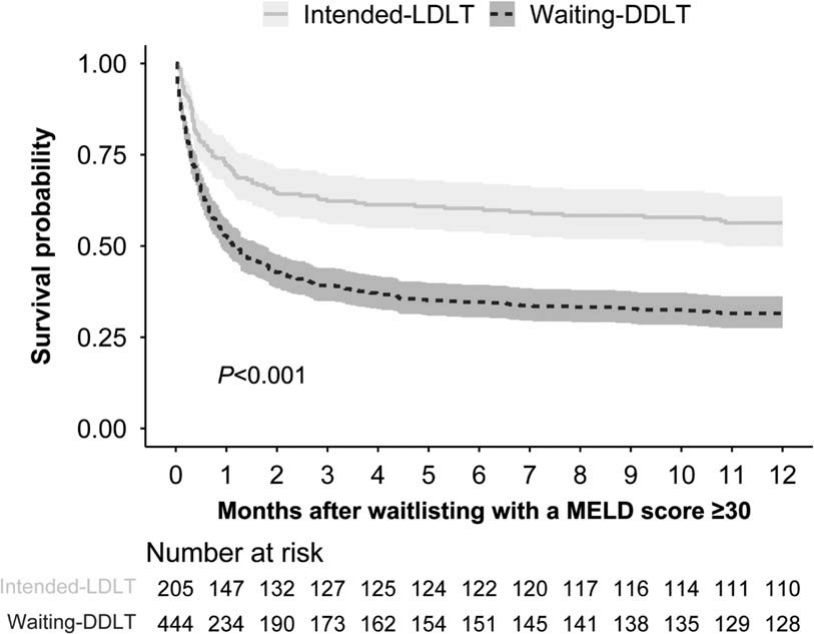
Comparison of survival curves after waitlisting for the Intended-LDLT versus Waiting-DDLT groups of patients with a MELD score greater than or equal to 30. DDLT, deceased-donor liver transplantation; LDLT, living-donor liver transplantation; MELD, Model for End-stage Liver Disease.

During follow-up, death occurred in 95 patients in the intended-LDLT group and 128 patients in the waiting-DDLT group. The intended-LDLT group had markedly higher patient survival after waitlisting (*P*<0.001; Fig. 2). The 6-month and 12-month patient survival rates for the intended-LDLT group were 59.5 and 53.7%, whereas those for the waiting-DDLT group were 34.0 and 28.8%. Multivariable Cox regression analysis revealed that LDLT intention was independently associated with a decreased risk of patient mortality after waitlisting [hazard ratio (HR), 0.62; 95% CI, 0.48–0.79; *P*<0.001; Table 2]. Factors associated with increased all-cause mortality were older age (HR, 1.03; 95% CI, 1.02–1.04; *P*<0.001), HCC (HR, 1.74; 95% CI, 1.40–2.17; *P*<0.001), organ failure involving the brain (HR, 2.01; 95% CI, 1.58–2.63; *P*<0.001), HRS (HR, 1.30; 95% CI, 1.06–1.60; *P*=0.011), and cardiovascular disease (HR, 1.51; 95% CI, 1.02–2.23; *P*=0.040). Compared to blood type A or B, blood type AB was a protective factor for all-cause mortality (HR, 0.69; 95% CI, 0.48–0.99; *P*=0.042).

**Table 2:** Univariable and multivariable Cox regression analysis for survival after waitlisting in patients with a Model for End-stage Liver Disease score greater than or equal to 30.

	Univariable		Multivariable	
Variables	HR (95% CI)	*P*	HR (95% CI)	*P*
Intended-LDLT (vs. Waiting-DDLT)	0.50 (0.39–0.63)	<0.001	0.62 (0.48–0.79)	<0.001
Age	1.03 (1.02–1.04)	<0.001	1.03 (1.02–1.04)	<0.001
Sex, male	1.03 (0.83–1.28)	0.782		
ABO type (vs. A or B)				
* *AB	0.77 (0.54–1.11)	0.165	0.69 (0.48–0.99)	0.042
* *O	1.17 (0.94–1.45)	0.161	1.11 (0.90–1.38)	0.335
Alcoholic liver disease	0.74 (0.58–0.95)	0.016		
MELD score (vs. 30–34)				
* *35–39	1.08 (0.84–1.39)	0.533		
* *≥40	1.04 (0.74–1.46)	0.819		
Hepatocellular carcinoma	2.32 (1.89–2.85)	<0.001	1.74 (1.40–2.17)	<0.001
Liver failure type (vs. ACLF 0)				
* *ACLF 1	2.12 (1.33–3.39)	0.002		
* *ACLF 2	1.28 (0.85–1.93)	0.232		
* *ACLF 3	2.30 (1.52–3.49)	<0.001		
* *ALF	1.14 (0.69–1.89)	0.612		
Hepatorenal syndrome	1.52 (1.24–1.85)	<0.001	1.30 (1.06–1.60)	0.011
Organ failure, brain	2.15 (1.67–2.77)	<0.001	2.04 (1.58–2.63)	<0.001
Organ failure, circulatory system	1.52 (1.22–1.90)	<0.001		
Organ failure, respiratory system	1.74 (1.31–2.32)	<0.001		
Cardiovascular disease	1.79 (1.21–2.64)	0.004	1.51 (1.02–2.23)	0.040
ICU stay before waitlisting	1.43 (1.14–1.79)	0.002		
MELD score increase in the prior 1 month, *(vs. <15)*				
* *≥15	1.44 (1.09–1.92)	0.011		
* *Initial score≥30	0.74 (0.58–0.94)	0.014		
Sepsis	1.37 (1.02–1.85)	0.037		
Pneumonia	1.51 (1.08–2.10)	0.017		

ACLF, acute-on-chronic liver failure; ALF, acute liver failure; CI, confidence interval; DDLT, deceased-donor liver transplantation; HR, hazard ratio; LDLT, living-donor liver transplantation; MELD, Model for End-stage Liver Disease.

### Subgroup analysis: according to MELD score groups

On the day of waitlisting, patients were divided into three subgroups based on their MELD score: 30–34, 35–39, and greater than or equal to 40. In the 30–34 MELD score subgroup, the intended-LDLT group had a significantly higher 1-year survival rate than the waiting-DDLT group (55.8 vs. 27.7%, *P*<0.001; Supplemental Digital Content 3, http://links.lww.com/JS9/A844). The intended-LDLT group also had a higher 1-year survival rate than the waiting-DDLT group in the 35–39 MELD score subgroup (53.5 vs. 25.8%, *P*=0.004). However, 1-year survival did not differ between intended-LDLT and waiting-DDLT groups in patients with a MELD score greater than or equal to 40 (41.7 vs. 42.2%, respectively, *P*=0.692).

### Information at the time of liver transplantation

Excluding the 25 patients who were removed from the waitlist, 120 patients underwent LDLT, 170 patients underwent DDLT, and 334 patients remained on the waitlist. At the time of LT, age, male sex, recipient BMI, pretransplantation MELD score, HCC, time from MELD score of 30 to LT, simultaneous kidney transplantation, donor male sex, and donor BMI were comparable in both the LDLT and DDLT group (Supplemental Digital Content 4, http://links.lww.com/JS9/A845). Compared to the DDLT group, the LDLT group showed several notable differences. They had a lower number of patients admitted to the intensive care unit (15.8 vs. 25.9, *P*=0.044), younger donor age (32.8±11.4 years vs. 48.4±14.8 years, *P*<0.001), lower levels of graft steatosis (16.1 vs. 33.1%, *P*=0.002), lower volume of red blood cells transfused during surgery (2400 ml vs. 2700 ml, *P*=0.027), shorter cold ischemic time (132 min vs. 376 min, *P*<0.001) and longer total operation time (655 min vs. 502 min, *P*<0.001). In terms of disease severity, the LDLT group generally exhibited lower MELD scores (*P*=0.192) and had a shorter duration of stay in the ICU (*P*=0.192) compared to the DDLT group. Within the LDLT group, the majority of grafts were right lobe grafts (95.8%), followed by left lobe grafts (3.4%), and right anterior section graft (0.8%). Additionally, we calculated the donor risk indices for the DDLT group. The median of the donor risk index was 1.8 with the IQR of 1.7–2.2.

### Post-transplantation recipient outcomes

As shown in Table 3, the in-hospital mortality rate was marginally lower in the LDLT group than in the DDLT group (17.5 vs. 27.1%, *P*=0.066). The 1-year mortality rate and duration of hospital stay after LT were comparable between the two groups. Major complications occurred in 33.1% of patients who underwent LDLT and 19.4% of patients who underwent DDLT; this difference was statistically significant (*P*=0.013). Biliary leakage (11.9 vs. 4.7%, *P*=0.043) and biliary stricture (27.1 vs. 10.0%, *P*<0.001) occurred more frequently in the LDLT group than in the DDLT group. The rates of complications involving the hepatic artery, portal vein, hepatic vein, or inferior vena cava were similar between groups. The rates of rejection within 1-year were also not significantly different between groups (LDLT vs. DDLT: 25.4 vs. 19.4, *P*=0.285). Although Kaplan–Meier survival analysis demonstrated superior survival after LDLT, compared to DDLT (*P*=0.044; Supplemental Digital Content 5, http://links.lww.com/JS9/A846), LDLT was not independently associated with a decreased risk of patient death on multivariable Cox regression analysis (after adjusting for age, cardiovascular disease, pretransplantation MELD score, HCC, donor age, donor BMI, and graft microvesicular steatosis) [HR for mortality of LDLT (vs. DDLT), 1.86; 95% CI, 0.73–4.75; *P*=0.193; Supplemental Digital Content 6, http://links.lww.com/JS9/A847).

**Table 3:** Post-transplantation outcomes.

Variables	LDLT (*n*=120)	DDLT (*n*=170)	*P*
Recipient			
* *In-hospital mortality	21 (17.5)	46 (27.1)	0.066
* *Death within 1-year	27 (22.5)	55 (32.4)	0.085
* *Hospital stay, days	26 (20–38)	33 (20–51)	0.085
Major complications (grade ≥3)			
* *Biliary leakage	14 (11.9)	8 (4.7)	0.043
* *Biliary stricture	32 (27.1)	17 (10.0)	<0.001
* *Hepatic artery complication	5 (4.2)	3 (1.8)	0.373
* *Portal vein complication	5 (4.2)	4 (2.4)	0.576
* *Hepatic vein or IVC complication	2 (1.7)	7 (4.1)	0.413
* *Total	39 (33.1)	33 (19.4)	0.013
* *Rejection within 1-year	30 (25.4)	33 (19.4)	0.285
Living-donor			
* *Mortality	0		
* *Hospital stay, days	12 (10–14)		
* *Readmission within 6 months	4 (3.3)		
Major complications (grade ≥3)			
* *Biliary leakage	3 (2.5)		
* *Biliary stricture	1 (0.8)		
* *Intestinal obstruction	1 (0.8)		
* *Total	5 (4.2)		

Data are presented as number (percentage) or median (interquartile range).

DDLT, deceased-donor liver transplantation; IVC, inferior vena cava; LDLT, living-donor liver transplantation.

Patients who remained on the waitlist (waitlist-only group) had a 30-day survival rate of 37.7% and a 1-year survival rate of 13.2%, which were markedly inferior to those of the LDLT and DDLT groups (Supplemental Digital Content 7, http://links.lww.com/JS9/A848). Additionally, we compared the characteristics of those who survived and those who deceased in the waitlist-only group (Supplemental Digital Content 8, http://links.lww.com/JS9/A849). Compared to survived patients, deceased patients were more likely to be older (44.5 vs. 57.0, *P*<0.001), have hepatitis B or C (*P*<0.001), higher grades of ACLF (*P*<0.001), HCC (*P*<0.001), and rapid increase of MELD score during a month (*P*<0.001).

### Living-donor outcomes

The median length of hospital stay for living donors was 12 days (IQR, 10–14). The readmission rate within 6 months was 3.33%. Biliary leakage (1.66%), acute pancreatitis (0.83%), and intestinal obstruction (0.83%) were the reasons for readmission. The total rate of major complications was 4.17% in living donors and was subdivided into biliary leakage (2.5%), biliary stricture (0.83%), and intestinal obstruction (0.83%).

### Sensitivity analysis

IPTW was conducted to adjust for selection bias. Many covariates, including age, HCC, HRS, CKD, and sepsis, were adjusted with IPTW (Supplemental Digital Content 9, http://links.lww.com/JS9/A850). Kaplan–Meier analysis was performed to validate the survival benefit of LDLT from the waitlisting day. After IPTW, the intended-LDLT group continued to show superior patient survival, compared to the waiting-DDLT group (Supplemental Digital Content 10, http://links.lww.com/JS9/A851).

## Discussion

Compared to awaiting DDLT, intended-LDLT was independently associated with decreased mortality in patients with a MELD score greater than or equal to 30. Among patients who ultimately underwent transplantation surgery, LDLT and DDLT recipients had comparable patient survival, despite LDLT being associated with a higher rate of surgical complications. From a living-donor perspective, there was no mortality, and only 4.17% of patients experienced major complications (Clavien–Dindo grade ≥3). Other authors have recommended against proceeding with LDLT in patients with a high MELD score^11–13^, but our results demonstrated that even patients with a MELD score greater than or equal to 30 had generally similar outcomes after LDLT, compared to DDLT. Thus, if an eligible living-donor is available, LDLT can improve patient survival in patients with a MELD score greater than or equal to 30.

In this study, the majority of independent risk factors for all-cause mortality after waitlisting were comparable to those identified previously. It is well-established that elderly recipients^28,29^, altered mental status^30^, history of HRS^31–34^, and cerebrovascular disease^35^ are associated with a poorer prognosis after intention of LDLT. Interestingly, neither sepsis nor ALF was an independent risk factor of mortality in our study. Contrary to the conventional belief that infection prior to LT is a poor prognostic factor for LT, recent studies have demonstrated that, if treated, bacterial infections have little effect on mortality^36–38^. LDLT may therefore be an option for patients with a high MELD score, infection, and organ failure. It is well known that LT for ALF has a lower survival rate than LT for other indications^39,40^. However, in our waitlist population, survival after waitlisting was comparable between patients with ALF and those with ACLF grade 0. This result likely reflects the allocation system in South Korea. Patients with ALF (such as fulminant hepatitis) have immediate allocation priority, whereas patients with ACLF may be required to wait until their MELD score is high enough to be allocated to a deceased-donor liver.

In subgroup comparisons of patient survival by MELD score on the day of waitlisting, intended-LDLT patients with a MELD score of 30–34 or 35–39 had a higher patient survival rate than Waiting-DDLT patients. However, there was no significant difference in survival between these groups in patients with a MELD score greater than or equal to 40. This raises the question of whether LDLT should be avoided in patients with a MELD score greater than or equal to 40. However, our result may be attributed to an insufficient number of patients in this subgroup or the wide range of MELD scores in this subgroup^21^.

A significantly lower proportion of patients undergoing LDLT were admitted to the ICU at the time of transplantation compared to those undergoing DDLT. Additionally, although not statistically significant, the duration of ICU stays for 10 days or longer prior to transplantation was shorter in the LDLT group than the DDLT group. This observation can be considered as an advantageous aspect of LDLT, as it allows for a reduced waiting period for LT in patients with a MELD score greater than or equal to 30. Similarly, grafts procured from living donors were more likely to exhibit superior quality compared to those obtained from deceased donors, as LDLT necessitates a thorough evaluation of potential donors. In this study, the donors were younger and had a lower rate of greater than 10% steatosis in the LDLT group, and the cold ischemic time was also shorter in this group. However, the procedure is much more technically challenging for LDLT than for DDLT^41^ because of the smaller vessel and duct diameters with partial grafts, the possibility of multiple bile duct anastomoses, and the reconstruction of hepatic outflow. This was reflected in the longer operation time for LDLT than for DDLT. It was also reflected in the higher complication rate for LDLT, with most complications being related to the biliary system; nonbiliary complications did not differ between the LDLT and DDLT groups. Combining all these factors yielded slightly better patient survival after actual LDLT on Kaplan–Meier analysis (*P*=0.044), but LDLT was not independently associated with improved survival after adjusting for MELD score and other confounders on multivariable analysis. Rates of in-hospital mortality and death within 1-year were lower after LDLT than after DDLT, but the differences between groups did not reach statistical significance.

In relation to patients in the waitlist-only group, our observations indicate that they had markedly inferior patient survival compared to patients who underwent either type of LT. Particularly, patients with older age, hepatitis B or C infection, advanced ACLF grade, HCC, and a rapid increase in MELD score within a month seemed to be more vulnerable to waitlist mortality. Meanwhile, South Korea is confronted with a scarcity of organ donations, leading to a low rate of DDLT, about 25–30% of total LT (Supplemental Digital Content 11, http://links.lww.com/JS9/A852). Although there has been a recent decrease in the average waiting time to ~230 days, this can mainly be attributed to the fact that only patients with an exceptionally high MELD score are being allocated deceased-donor livers, and they have not been on the waitlist for a significant period of time. Therefore, waitlisted patients exhibiting these characteristics with a MELD score greater than or equal to 30 may require LDLT with greater urgency. However, it is important to note that this aspect falls outside the scope of our study and should be interpreted with caution.

In the study by Wong *et al*.^19^ examining the LDLT in patients with a high MELD score, the authors used a MELD score of 25 as the cut-off value. In regions with a severe shortage of organs, such as South Korea; however, the likelihood of undergoing DDLT with a MELD score of 25–30 is exceedingly low. Because of variations in DDLT allocation processes across different regions, the potential survival benefits of LDLT in patients with a high MELD score should be validated in other LT settings. Furthermore, Wong *et al*. reported a wide range of waiting periods. If there is a significant time interval between waitlisting and the transplantation procedure, it may be challenging to accurately reflect the treatment intention and immortal time bias becomes a possible issue. To address this concern, we restricted our analysis to patients who underwent LT within 90 days from the date of waitlisting.

The major strengths of this study are its large sample size and prolonged follow-up period, as well as the comprehensive data analysis used to correct for statistical bias. However, it has some limitations. Because of its retrospective design, selection bias remains possible. Nonetheless, we performed multivariable Cox regression analysis and IPTW to address potential confounding factors. Our data were also obtained from a single experienced, high-volume transplant center, which may limit the generalizability of our results. Further studies considering the experience of other institutions are required.

In conclusion, compared to waiting for DDLT, intended-LDLT was associated with a considerable survival benefit for patients with a MELD score greater than or equal to 30 in a region with a severe deceased organ shortage, with acceptable outcomes achieved in both recipients and donors. LDLT should be considered a feasible treatment option for patients with a MELD score greater than or equal to 30.

## Ethical approval

The institutional review board of Severance Hospital approved the study (4-2022-0913).

## Consent

Patient consent for this study was waived because of its retrospective design.

## Author contribution

SH.Y.: conception and design, acquisition of data, drafting and revising the article; DG.K.: conception and design, acquisition of data, statistical analysis, interpretation, drafting and revising the article; DJ.J.: conception and design, data interpretation, drafting the article; MY.K., HH.K., MC.C., EK.M., JG.L., MS.K.: study design, acquisition of data with analysis and interpretation.

## Conflicts of interest disclosure

The authors declare that they have no conflicts of interest.

## Research registration unique identifying number (UIN)

1. Name of the registry: CRIS (Clinical Research Information service).
2. Unique Identifying number or registration ID: 20230509-007.
3. Hyperlink to your specific registration (must be publicly accessible and will be checked): https://cris.nih.go.kr/cris/search/detailSearch.do?seq=24765&status=1&seq_group=24765&search_page=M

## Guarantor

Deok-Gie Kim, MD, Clinical Associate Professor, Department of Surgery, The Research Institute for Transplantation, Yonsei University College of Medicine Address: 50-1 Yonsei-ro, Seodaemun-gu, Seoul 03722, South Korea. Tel: +82 2 2228 2100, fax: +82 2 313 8289, E-mail: mppl01@yuhs.ac

## Data availability statement

Datasets generated during the current study are available, upon reasonable request.

## Provenance and peer review

Not commissioned, externally peer-reviewed.

## Supplementary Material
