# Supplementary material for: Survival benefit of living-donor liver transplantation in patients with a model for end-stage liver disease over 30 in a region with severe organ shortage: a retrospective cohort study
Source: Int J Surg. 2023 Aug 10;109(11):3459–66. doi: 10.1097/JS9.0000000000000634 (PMC10651284; doi:10.1097/JS9.0000000000000634)
Supplement: Supplementary file 2 [file js9-109-3459-s002.docx]

**Supplemental Digital Content 2. Causes of LDLT withdrawal.**

| **Causes of LDLT hold** | **Number (%)** |
| --- | --- |
| Deteriorating health status | 25 (27.2) |
| Improved patients’ condition | 9 (9.8) |
| Receive DDLT | 20 (21.7) |
| Incompatible donor | 24 (26.1) |
| Donation withdrawal | 6 (6.5) |
| Refused by KONOS | 2 (2.2) |
| Others | 6 (6.5) |

LDLT, living-donor liver transplantation, DDLT, deceased-donor liver transplantation; KONOS, Korean network for organ sharing,
