# Supplementary material for: Survival benefit of living-donor liver transplantation in patients with a model for end-stage liver disease over 30 in a region with severe organ shortage: a retrospective cohort study
Source: Int J Surg. 2023 Aug 10;109(11):3459–66. doi: 10.1097/JS9.0000000000000634 (PMC10651284; doi:10.1097/JS9.0000000000000634)
Supplement: Supplementary file 3 [file js9-109-3459-s003.pptx]

## Slide 1
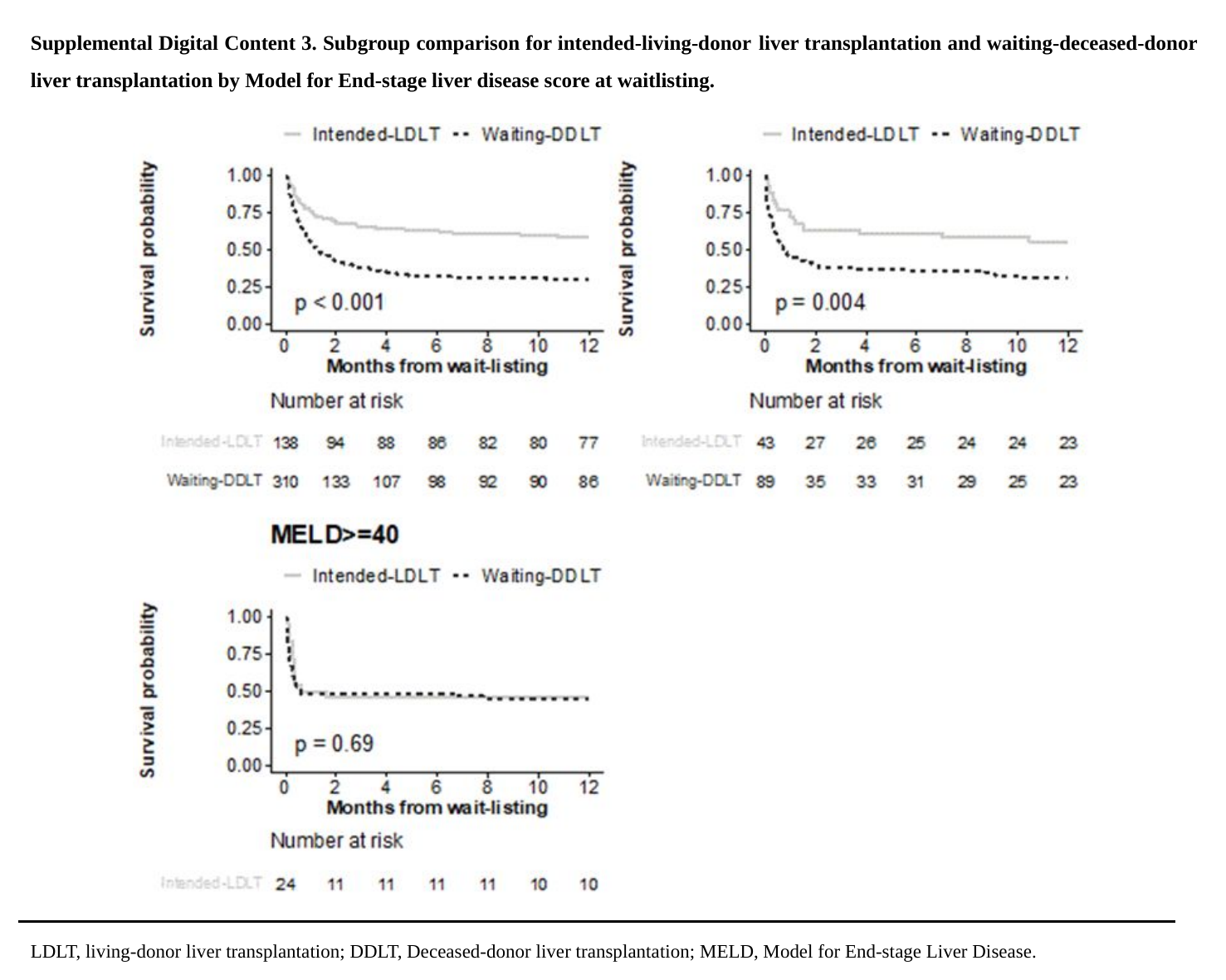

Supplemental Digital Content 3. Subgroup comparison for intended-living-donor liver transplantation and waiting-deceased-donor liver transplantation by Model for End-stage liver disease score at waitlisting.
LDLT, living-donor liver transplantation; DDLT, Deceased-donor liver transplantation; MELD, Model for End-stage Liver Disease.
