# Supplementary material for: Survival benefit of living-donor liver transplantation in patients with a model for end-stage liver disease over 30 in a region with severe organ shortage: a retrospective cohort study
Source: Int J Surg. 2023 Aug 10;109(11):3459–66. doi: 10.1097/JS9.0000000000000634 (PMC10651284; doi:10.1097/JS9.0000000000000634)
Supplement: Supplementary file 4 [file js9-109-3459-s004.docx]

**Supplemental Digital Content 4. Information at the time of liver transplantation.**

| **Variables** | **LDLT**  **(n = 120)** | **DDLT**  **(n = 170)** | ***P*** |
| --- | --- | --- | --- |
| Age, year | 50.8 ± 9.7 | 51.1 ± 11.9 | 0.809 |
| Sex, male | 79 (65.8) | 117 (68.8) | 0.683 |
| Body mass index | 24.0 (21.6-25.9) | 23.7 (21.8-26.5) | 0.845 |
| Pretransplantation MELD score | 33.5 ± 4.0 | 34.6 ± 5.5 | 0.065 |
| Hepatocellular carcinoma | 20 (16.7) | 34 (20.0) | 0.572 |
| Time from MELD 30 to transplantation, days | 10 (3-24) | 9 (5-17) | 0.970 |
| ICU stay before transplant, patients | 19 (15.8) | 44 (25.9) | 0.044 |
| ICU stay before transplant, days |  |  | 0.192 |
| *0* | 92 (76.7) | 123 (72.4) |  |
| *1-2* | 2 (1.7) | 2 (1.2) |  |
| *3-9* | 15 (12.5) | 14 (8.2) |  |
| *10-* | 11 (9.2) | 31 (18.2) |  |
| Simultaneous kidney transplantation | 1 (0.8) | 5 (2.9) | 0.421 |
| Donor age | 32.8 ± 11.4 | 48.4 ± 14.8 | <0.001 |
| Donor sex, male | 74 (62.7) | 109 (64.1) | 0.905 |
| Donor body mass index | 22.7 (21.5-25.2) | 23.0 (21.0-25.0) | 0.992 |
| Donor risk index | - |  |  |
| *1.0 < DRI ≤ 2.0* |  | 78 (45.9) |  |
| *2.0 < DRI ≤ 3.0* |  | 73 (42.9) |  |
| *3.0 < DRI* |  | 19 (11.2) |  |
| Graft types |  |  | 1.000 |
| *Whole liver* | 0 (0) | 170 (100) |  |
| *Right lobe* | 115 (95.8) | 0 (0) |  |
| *Left lobe* | 4 (3.4) | 0 (0) |  |
| *Right anterior section* | 1 (0.8) | 0 (0) |  |
| Graft steatosis ≥10% | 19 (16.1) | 56 (33.1) | 0.002 |
| Operation time, min | 655 (559-736) | 502 (420-603) | <0.001 |
| Cold ischemic time, min | 132 (109-168) | 376 (300-480) | <0.001 |
| RBC transfusion, mL | 2400 (1200-3900) | 2700 (1500-5100) | 0.027 |

Data are presented as numbers (percentage), or median (IQR)

LDLT, living-donor liver transplantation; DDLT, deceased-donor liver transplantation; RBC, red blood cell; MELD, Model for End-stage Liver Disease
