# Supplementary material for: Survival benefit of living-donor liver transplantation in patients with a model for end-stage liver disease over 30 in a region with severe organ shortage: a retrospective cohort study
Source: Int J Surg. 2023 Aug 10;109(11):3459–66. doi: 10.1097/JS9.0000000000000634 (PMC10651284; doi:10.1097/JS9.0000000000000634)
Supplement: Supplementary file 6 [file js9-109-3459-s006.docx]

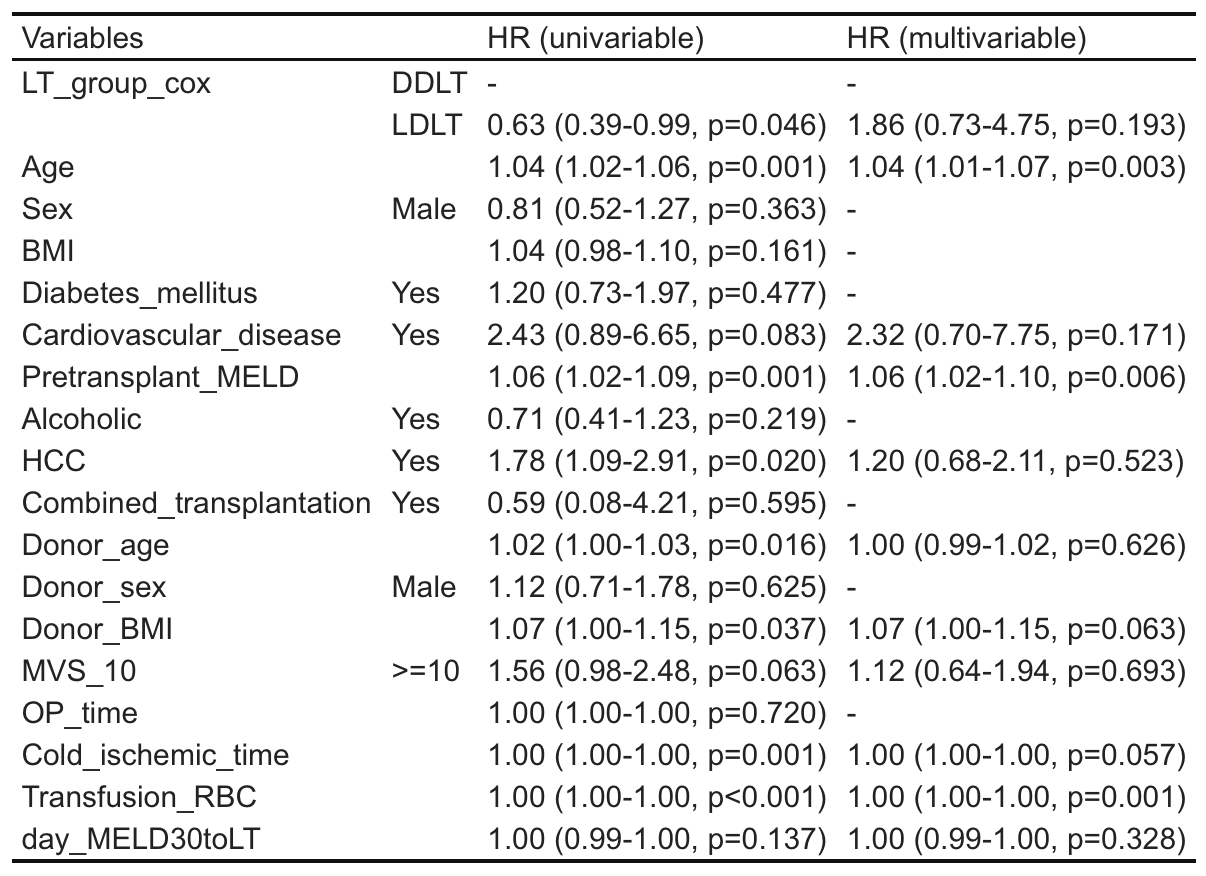
**Supplemental Digital Content 6. Univariable and Multivariable Cox regression analysis of patients who received LT for survival after transplantation in patients with a Model for End-stage Liver Disease score ≥30.**

HR, Hazard ratio; LT, liver transplantation; DDLT, deceased-donor liver transplantation; LDLT, living-donor liver transplantation; BMI, body mass index; MELD, Model for End-stage Liver Disease; HCC, hepatocellular carcinoma; MVS, microvesicular steatosis; OP, operation; RBC, red blood cell.
