# Supplementary material for: Survival benefit of living-donor liver transplantation in patients with a model for end-stage liver disease over 30 in a region with severe organ shortage: a retrospective cohort study
Source: Int J Surg. 2023 Aug 10;109(11):3459–66. doi: 10.1097/JS9.0000000000000634 (PMC10651284; doi:10.1097/JS9.0000000000000634)
Supplement: Supplementary file 7 [file js9-109-3459-s007.pptx]

## Slide 1
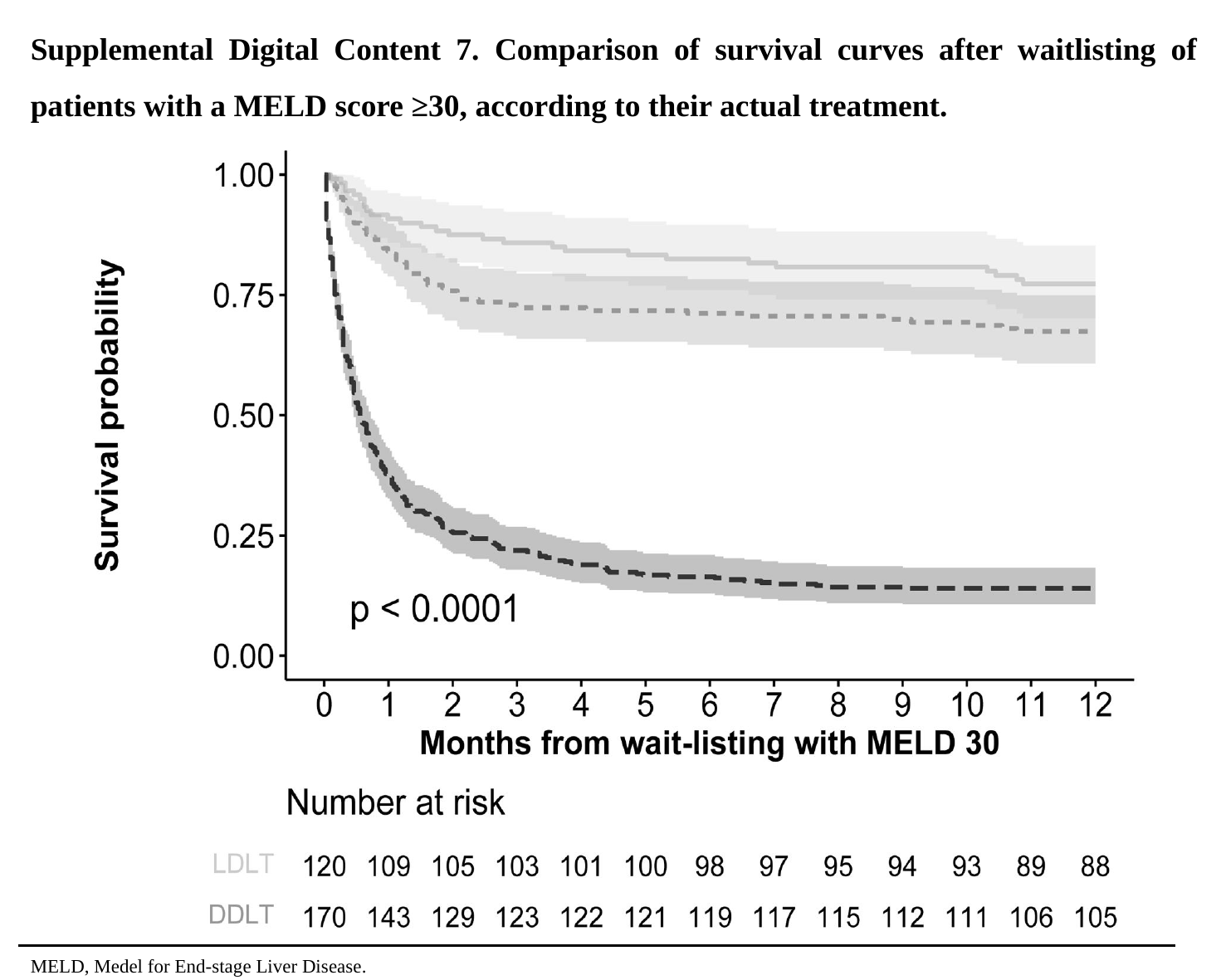

Supplemental Digital Content 7. Comparison of survival curves after waitlisting of patients with a MELD score ≥30, according to their actual treatment.
MELD, Medel for End-stage Liver Disease.
