# Supplementary material for: Survival benefit of living-donor liver transplantation in patients with a model for end-stage liver disease over 30 in a region with severe organ shortage: a retrospective cohort study
Source: Int J Surg. 2023 Aug 10;109(11):3459–66. doi: 10.1097/JS9.0000000000000634 (PMC10651284; doi:10.1097/JS9.0000000000000634)
Supplement: Supplementary file 8 [file js9-109-3459-s008.docx]

**Supplemental Digital Contents 8. Comparison between patients who survived vs. deceased in Waitlist-only group.**

| **Variables** | **Survived**  **(n=48)** | **Deceased**  **(n=286)** | ***P*** |
| --- | --- | --- | --- |
| Age, year | 44.5 (34.5–52.0) | 57.0 (49.0–63.0) | <0.001 |
| Sex, male | 36 (75.0) | 208 (72.7) | 0.879 |
| ABO type |  |  | 0.793 |
| *A or B* | 28 (58.3) | 167 (58.4) |  |
| *AB* | 5 (10.4) | 22 (7.7) |  |
| *O* | 15 (31.2) | 97 (33.9) |  |
| Underlying liver disease |  |  | <0.001 |
| *Alcoholic* | 15 (31.2) | 56 (19.6) |  |
| *HBV* | 18 (37.5) | 162 (56.6) |  |
| *HCV* | 1 (2.1) | 17 (5.9) |  |
| *HAV* | 7 (14.6) | 4 (1.4) |  |
| *Cryptogenic* | 5 (10.4) | 31 (10.8) |  |
| *Autoimmune* | 1 (2.1) | 9 (3.1) |  |
| *Other* | 1 (2.1) | 7 (2.4) |  |
| MELD score |  |  | 0.939 |
| *30–34* | 36 (75.0) | 208 (72.7) |  |
| *35–39* | 9 (18.8) | 57 (19.9) |  |
| *≥40* | 3 (6.2) | 21 (7.3) |  |
| Liver failure type |  |  | <0.001 |
| *ACLF 0* | 1 (2.1) | 19 (6.6) |  |
| *ACLF 1* | 4 (8.3) | 41 (14.3) |  |
| *ACLF 2* | 23 (47.9) | 122 (42.7) |  |
| *ACLF 3* | 8 (16.7) | 92 (32.2) |  |
| *ALF* | 12 (25.0) | 12 (4.2) |  |
| Milan criteria |  |  | <0.001 |
| -No HCC | 46 (95.8) | 159 (55.6) |  |
| -Within | 1 (2.1) | 70 (24.5) |  |
| -Above | 1 (2.1) | 57 (19.9) |  |
| Organ failures |  |  |  |
| *Brain* | 6 (12.5) | 52 (18.2) | 0.450 |
| *Coagulation* | 22 (45.8) | 137 (47.9) | 0.913 |
| *Circulation* | 7 (14.6) | 76 (26.6) | 0.110 |
| *Respiratory* | 4 (8.3) | 37 (12.9) | 0.508 |
| Hepatorenal syndrome | 30 (62.5) | 166 (58.0) | 0.673 |
| Chronic kidney disease | 4 (8.3) | 28 (9.8) | 0.958 |
| Cardiovascular disease | 2 (4.2) | 22 (7.7) | 0.566 |
| ICU stay before waitlisting, patients | 9 (18.8) | 68 (23.8) | 0.562 |
| MELD score increase in the prior 1 month |  |  | <0.001 |
| *<15* | 9 (18.8) | 79 (27.6) |  |
| *≥15* | 2 (4.2) | 74 (25.9) |  |
| *Initial score ≥30* | 37 (77.1) | 133 (46.5) |  |
| Sepsis | 8 (16.7) | 37 (12.9) | 0.637 |
| Pneumonia | 4 (8.3) | 30 (10.5) | 0.842 |

Data are presented as number (percentage) or median (interquartile range).

HBV, hepatitis B virus; HCV, hepatitis C virus; HAV, hepatitis A virus; MELD, Model for End-stage Liver Disease; ACLF, acute-on-chronic liver failure; ALF, acute liver failure; HCC, hepatocellular carcinoma; ICU, intensive care unit.
