# Supplementary material for: Survival benefit of living-donor liver transplantation in patients with a model for end-stage liver disease over 30 in a region with severe organ shortage: a retrospective cohort study
Source: Int J Surg. 2023 Aug 10;109(11):3459–66. doi: 10.1097/JS9.0000000000000634 (PMC10651284; doi:10.1097/JS9.0000000000000634)
Supplement: Supplementary file 9 [file js9-109-3459-s009.pptx]

## Slide 1
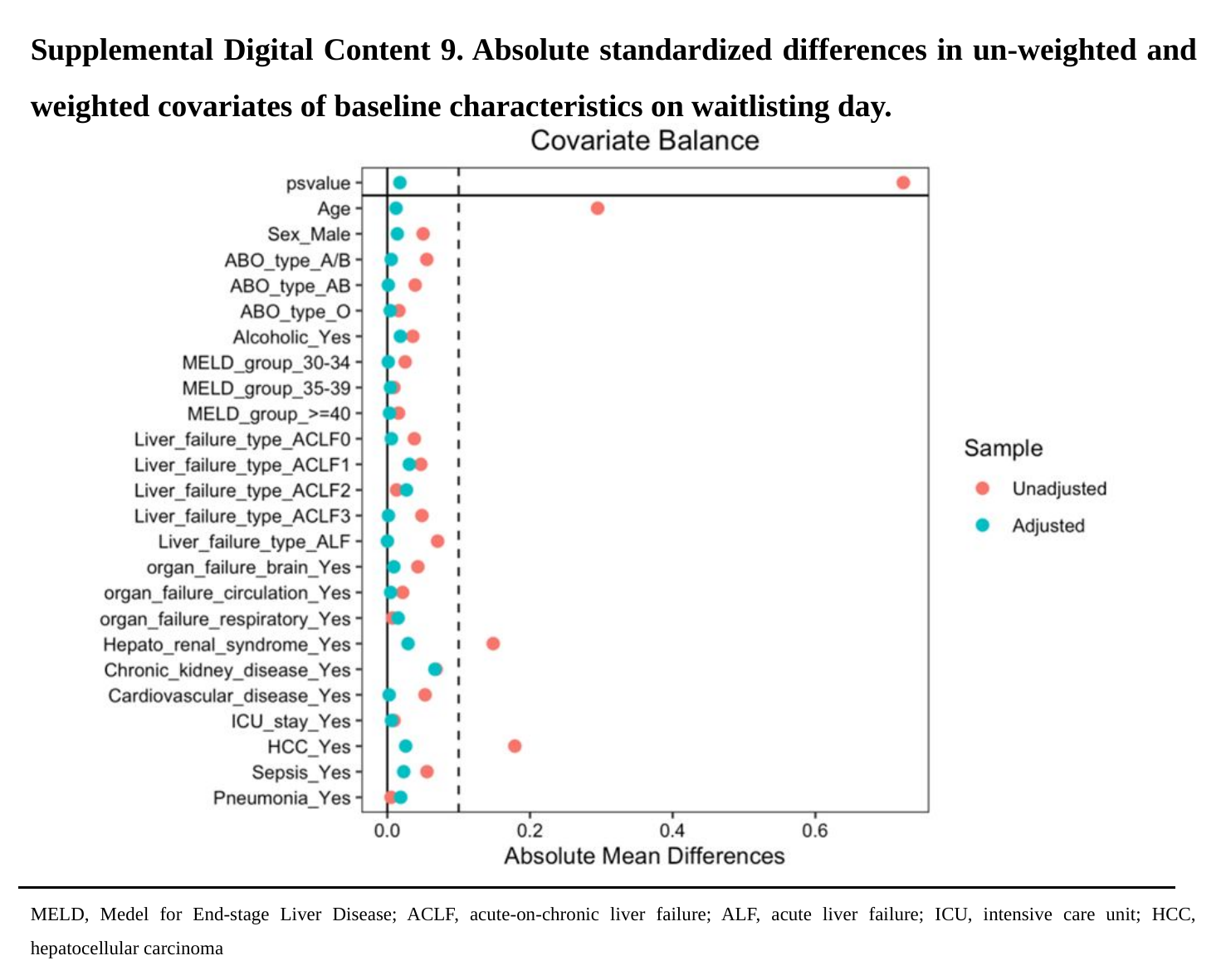

Supplemental Digital Content 9. Absolute standardized differences in un-weighted and weighted covariates of baseline characteristics on waitlisting day.
MELD, Medel for End-stage Liver Disease; ACLF, acute-on-chronic liver failure; ALF, acute liver failure; ICU, intensive care unit; HCC, hepatocellular carcinoma
