# Supplementary material for: Survival benefit of living-donor liver transplantation in patients with a model for end-stage liver disease over 30 in a region with severe organ shortage: a retrospective cohort study
Source: Int J Surg. 2023 Aug 10;109(11):3459–66. doi: 10.1097/JS9.0000000000000634 (PMC10651284; doi:10.1097/JS9.0000000000000634)
Supplement: Supplementary file 10 [file js9-109-3459-s010.pptx]

## Slide 1
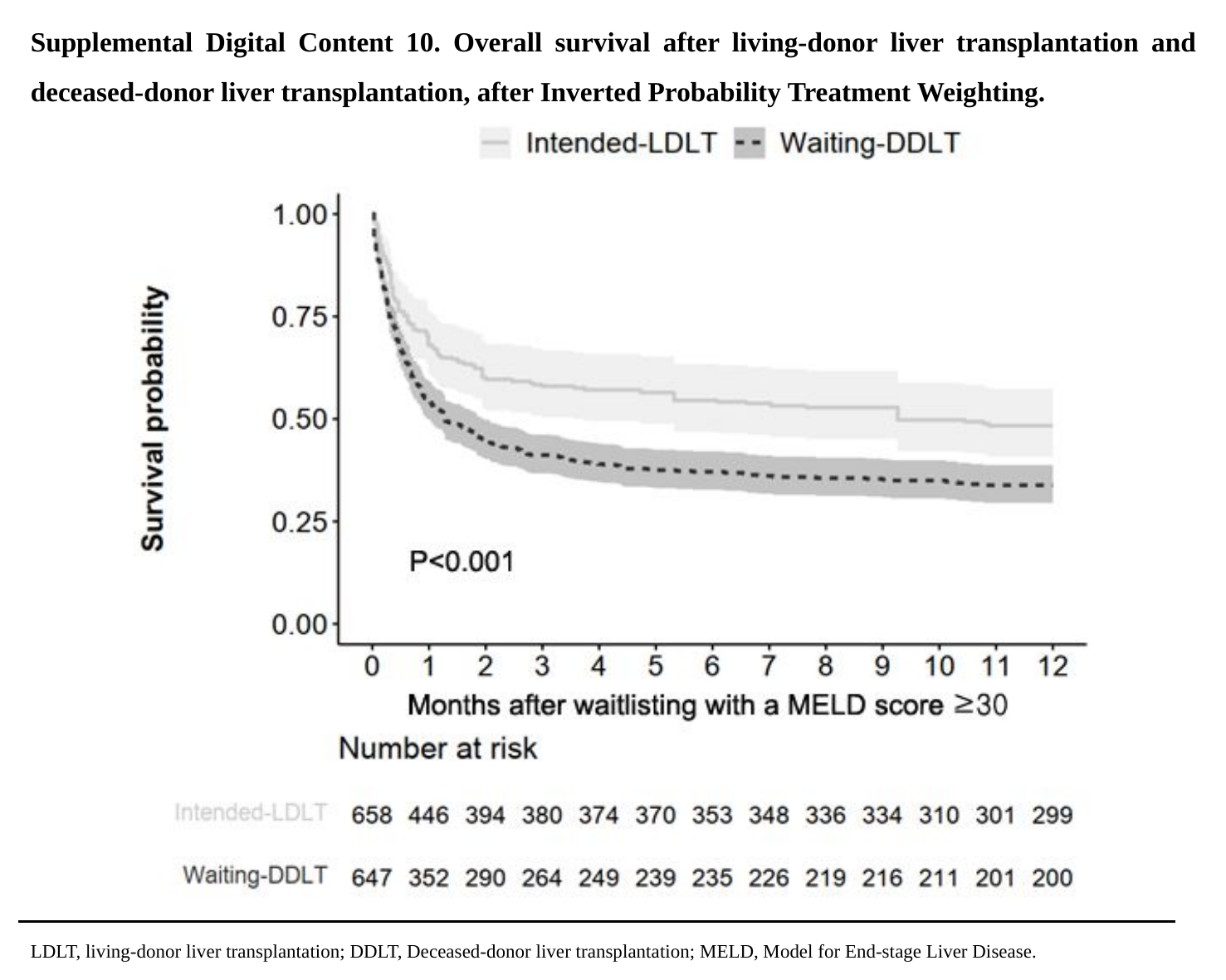

Supplemental Digital Content 10. Overall survival after living-donor liver transplantation and deceased-donor liver transplantation, after Inverted Probability Treatment Weighting.
LDLT, living-donor liver transplantation; DDLT, Deceased-donor liver transplantation; MELD, Model for End-stage Liver Disease.
