# Supplementary material for: Survival benefit of living-donor liver transplantation in patients with a model for end-stage liver disease over 30 in a region with severe organ shortage: a retrospective cohort study
Source: Int J Surg. 2023 Aug 10;109(11):3459–66. doi: 10.1097/JS9.0000000000000634 (PMC10651284; doi:10.1097/JS9.0000000000000634)
Supplement: Supplementary file 11 [file js9-109-3459-s011.pptx]

## Slide 1
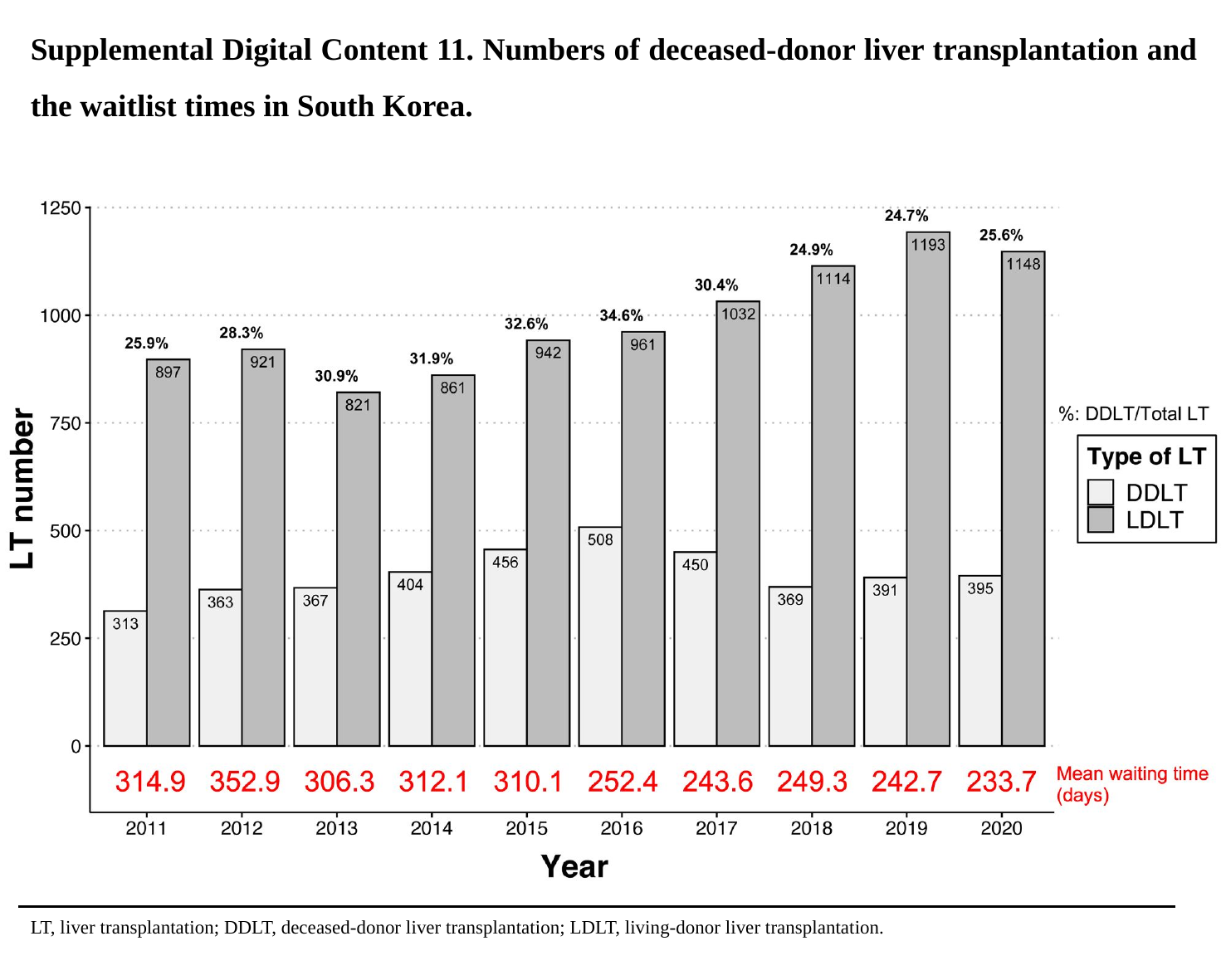

Supplemental Digital Content 11. Numbers of deceased-donor liver transplantation and the waitlist times in South Korea.
LT, liver transplantation; DDLT, deceased-donor liver transplantation; LDLT, living-donor liver transplantation.
